# Study on layout optimization of sewage outfalls: a case study of wastewater treatment plants in Xiamen

**DOI:** 10.1038/s41598-021-97756-9

**Published:** 2021-09-15

**Authors:** Cui Wang, Zhouhua Guo, Qingsheng Li, Jing Fang

**Affiliations:** Third Institute of Oceanography, Ministry of Natural Resources, Xiamen, 361005 China

**Keywords:** Environmental impact, Physical oceanography

## Abstract

In order to protect the offshore environment and strengthen the comprehensive rectification of sewage outfalls, an evaluation method of regional sewage outfalls by combining the marine numerical simulation and comprehensive evaluation technology was constructed, considering the marine environmental capacity and the ecological impact of sewage discharge from outfalls on the marine eco-environment sensitive areas. Then the layout rationality of each outfall was evaluated and the discharge scale was optimized with a case study of existing sewage outfalls in Xiamen. The results show that, the comprehensive evaluation score of Yundang outfall was 3 points in 2025, evaluated as the outfall with irrational layout. In 2035, the comprehensive evaluation scores of Fenglin and Dalipu outfalls were 3 and 2 points respectively, evaluated as the outfall with irrational discharge scale. It is suggested to control the scale of expansion or increase the reclaimed water reuse rate in Jimei and Gaoqi Wastewater Treatment Plants. This method has enriched the evaluation system for layout optimization of sewage outfalls, providing scientific supports for comprehensive improvement of sewage outfalls and marine environmental management.

## Introduction

The ocean is the ultimate destination of terrigenous pollutants^1^. Discharging the domestic sewage and industrial wastewater meeting the discharge standards into the ocean after centralized treatment is an economical strategy to utilize the marine environment and relieve the offshore environmental pressure^2,3^, which is also one of the main ways of sewage discharge in the coastal areas^4^. The ocean can treat some pollutants through self-purification capacity. However, due to the constraints on objective factors such as the geographical position, hydrodynamic conditions and physicochemical characterization of pollutants, the marine environmental capacity is limited^5^, which cannot absorb all the pollutants dicharged into the ocean. The unreasonable layout of sewage outfalls can cause serious environmental problems in the surrounding sea areas^4,6^.

In recent years, with the acceleration of urbanization process and the rapid development of social economy in the coastal areas, the pollutants directly or indirectly discharged into the sea areas have been increasing. The coastal area has been suffering from the pollutants discharge intensity beyond its self-purification capacity for a long time, leading to the increasing environmental pollution^7,8^. According to the Report on the State of the Marine Ecological Environment in China in 2019, the environmental quality of the sea areas near the offshore outfalls in China was generally poor, and over 90% of the water quality failed to meet the standard of environmental protection in the marine functional zones. Marine environmental pollution is still the dominant factor that restricts the marine economic development and ecological security^9,10^.

The model is a useful tool to select and determine the optimal sewage outlet. Foreign scholars calculated the initial dilution ability mainly by adopting the far-field numerical circulation model^11^, Gaussian-vortex model^12^, VISFJET^13^ and near-field pollutant diffusion model CORMIX, which was used as the main reference index for the setting of outfalls. Later, with the development of computers, the three-dimentional models (Delft3D^14^, EFDC^15^, Plumes-UM3) were usually used for hydrodynamic coupling with plume models^16^ to simulate the initial dilution ability and transportation of pollutant discharged from outfalls and select the outfalls. In addition, Minjeong et al.^4^ predicted the spatiotemporal variation of *Escherichia coli* and antibiotic resistance genes with different locations based on the EFDC model, thereby determining the effective design of marine outfalls.

With the construction of the coastal city sewage discharge project, the research on the selection and planning of the sewage outfalls began in the mid-1990s in China. The research methods generally include the numerical simulation model and comprehensive index system. Firstly, the numerical calculation method is used to simulate the influence of hydrodynamic environment, and then select the best discharge scheme. For example, the particle movement trajectory was simulated by the Lagrange particle tracking model^17,18^. The influence range of pollutants was calculated by the pollutant diffusion model^19,20^. The optimization position was determined by comparing the distance of particle trajectory, the extent of pollutant diffusion and/or the environment capacity^21–23^. The marine hydrodynamic factors have been fully taken into consideration in the numerical method, which is in agreement with the actual situation of marine pollutant diffusion. However, other factors such as engineering cost were ignored in the selection of location. Secondly, the comprehensive index system was established to determine the best one by ranking the comprehensive score of different discharge outfalls. For example, the range of COD pollutant area, the sediment change and engineering cost were considered as the indicators in the study of Yangkou Port sewage site selection^24^. Additional indicator system also includes the aspects of environment (mixing zone area^25^, environmental capacity^26^, self-purification capacity^27^, the distance from marine eco-environment sensitive area), natural conditions (topographic slope, geomorphological features)^28^. The index system method considered comprehensive indictors to evaluate the sewage outfall. However, the obvious disadvantage of this method is high requirement for basic data and poor practicability due to the complexity of the evaluation system^29^.

In the whole, the previous studies mainly focused on the selection and planning sewage outfalls for a new specific sewage discharge project to determine the best one. There is a lack of research on the resonable evaluation of the existing outfalls and optimization of the future discharge scale from the regional perspective^30^. In order to fully promote the construction of marine ecological civilization demonstration zone^31,32^, advance the rational utilization of sea area environmental capacity and the harmonious development of marine economy, a optimization evaluation method of regional sewage outfalls by combining the marine numerical simulation and comprehensive evaluation technology was constructed to evaluate the reasonableness location and optimize discharge scale of the existing outfalls. This study not only can effectively deal with terrigenous pollutants and reduce environmental pollution, but also can effectively reduce the risk of marine ecological environment^33^, which can provide decision support for the management of sewage outlet into the sea and Marine environmental protection.

## Study areas and methods

### Study areas

**Figure 1:**
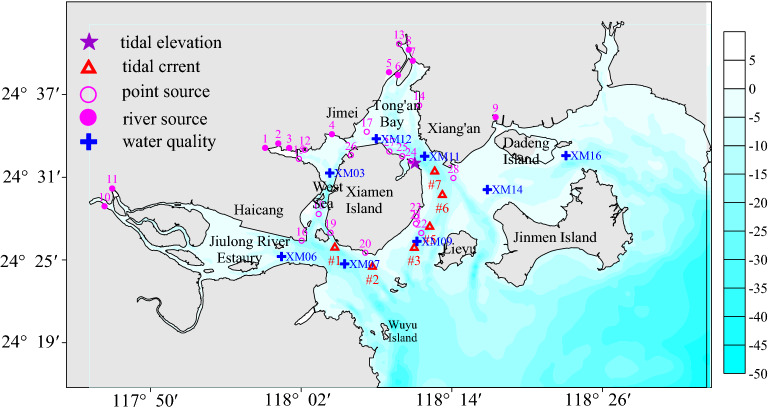
Topography of XMB and location map of stations. #1-#6 indicate the locations of the tidal current stations. 1–11 show the location of river sources and 12–28 show the location of point sources discharged into the XMB. XM03, XM06, XM07, XM09, XM11, XM12, XM14 and XM16 represent the water quality verification stations. The figure was plotted with Surfer10 Software (https://www.goldensoftware.com/products/surfer).

**Figure 2:**
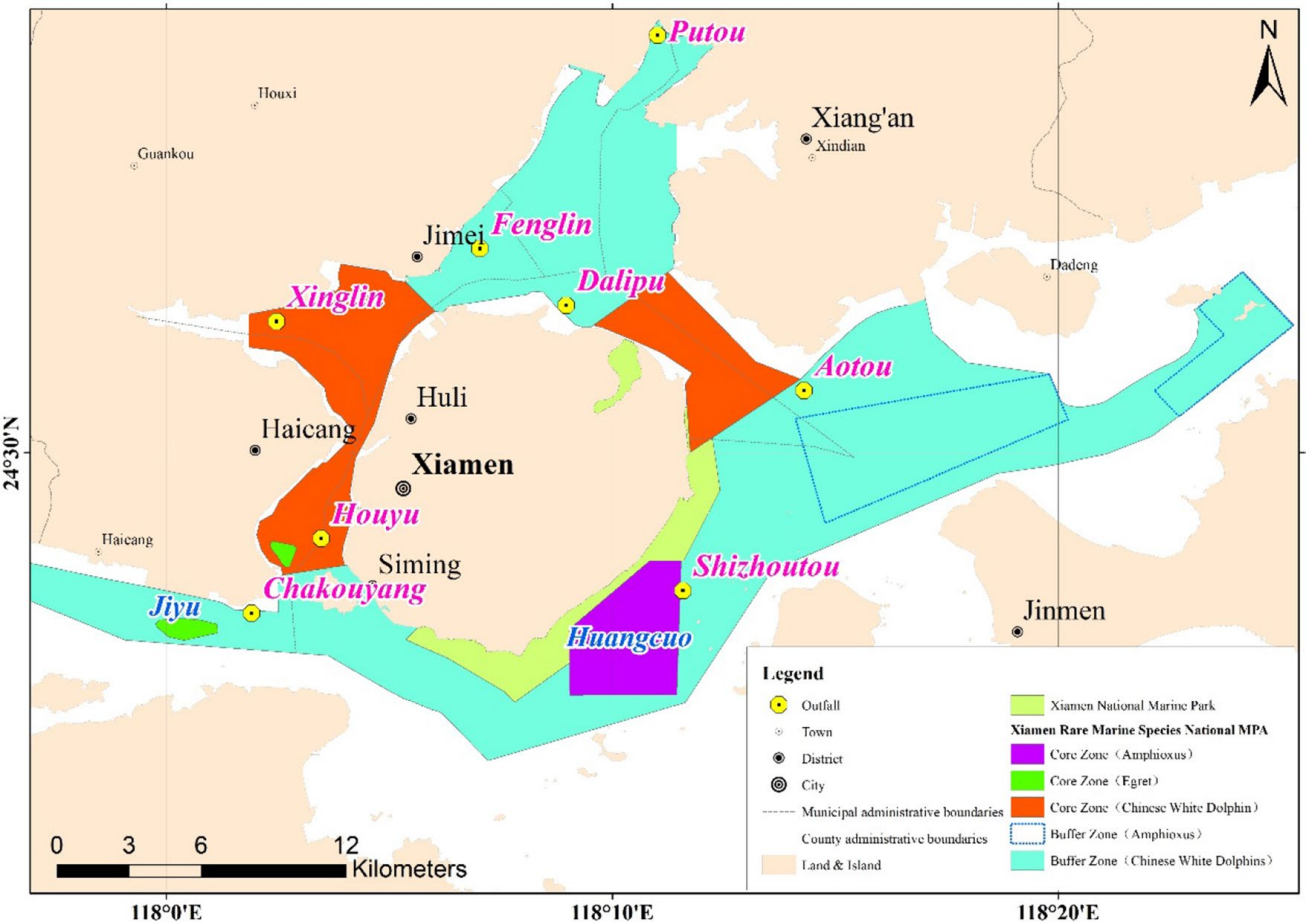
Location of pollutant discharge sea areas in Xiamen and marine eco-environment sensitive areas of XMB. The figure was drawn with ArcGIS software version 10.0 (https://www.arcgis.com/index.html).

Located in the southeast of Fujian Province, Xiamen Bay (XMB) is surrounded by islands such as Jinmen, Dadan, Erdan and Wuyu islands, all of which form into a natural barrier (Figs. 1, 2). XMB has a tortuous coastline with a total length of 194 km and a sea area of 390 km^2^. As a major Rare Marine Species National Nature Reserve with the Chinese White Dolphin, Lancelet and Egret wild animals, XMB is a semi-closed bay of great conservation value.

By 2020, there have been 5 wastewater treatment plants in Xiamen that directly discharged waster water into the sea, with a total discharge scale of 7.0 × 10^5^ t/d. The scale and discharge area of each wastewater plant are shown in Table 1 and Fig. 2. According to Study on Layout of Wastewater Treatment Plants and Treatment System Planning in Xiamen, Haicang, Qianpu, Jimei and Aotou wastewater plants will be expanded respectively. Meanwhile, Gaoqi plant is under construction. In the future, the total discharge scale will be increased from 1.43 × 10^6^ t/d in 2025 to 2.50 × 10^6^ t/d in 2035. With the development of Xiamen economy and the gradual improvement of sewage collection system, the amount of sewage entering the wastewater treatment plant has been increasing. Meanwhile, the remediation and management of sewage outlet into the sea has become the focus of environmental protection work at present in Xiamen. Therefore, the rationality of existing sewage outfalls layout and the optimization of the future discharge scale have become an urgent problem for each wastewater treatment plant in Xiamen.

**Table 1:** Discharge scale and discharge area of wastewater treatment plants in Xiamen.

No	Name	Discharge scale (× 10^4^ t/d)			Discharge standard	Name of discharge area
		2020	2025	2035		
1	Yundang plant	30	30	30	Grade 1 A	Houyu
2	Haicang plant	10	20	40	Grade 1 A	Chakouyang
3	Qianpu plant	20	50	50	Grade 1 A	Shizhoutou
4	Jimei plant	9	15	25	Class IV surface water	Fenglin
5	Aotou plant	1	8	55	Grade 1 A	Aotou
6	Gaoqi plant	0	20	50	Grade 1 A	Dalipu
	Total	70	143	250	–	–

### Evaluation methods

#### Principles of the evaluation system construction

It is important to establish a set of scientific and feasible evaluation index system for optimization of sewage outfalls. It is clearly stated in the Article 30 of the Marine Environment Protection Law of the People's Republic of China that, “the location of sewage outfalls shall be selected according to the marine functional zoning, seawater hydrodynamic conditions and relevant regulations”; “no new sewage outfalls shall be built in special protection areas such as the marine nature reserves, key fisheries areas and seaside scenic areas.” The principles of evaluation system construction should include:

1. Conform with the regulations, laws and the marine function zoning;
2. Good hydrodynamic conditions and strong self-cleaning ability;
3. The ecological impact of the sea area near the sewage outfalls should be reduced as far as possible;
4. Engineering feasibility and low construction cost.

Based on the above four principles and the objective of this study, we built in the optimization evaluation index system from the aspect of the hydrodynamic conditions and the ecological impact on the marine environment.

#### Selection of evaluation indicators

The evalation indicators were divided into two dimensions, which concluded the hydrodynamic conditions indicator and the ecological impact indicator on the marine environment.

1. Hydrodynamic conditions indicator

Terrigenous pollutants will transport and diffuse with the movement of seawater after being discharged into the sea. The diffusion mainly depends on the hydrodynamic conditions of the sea area near the sewage outfall. If the sea area near the sewage outfall has poor hydrodynamic conditions and water exchange capacity, pollutants will accumulate near the outfall, aggravating the pollution of the local sea areas and causing a series of marine ecological security problems^33^. Environmental capacity is a quantitative description of the comprehensive self-purification capacity of the sea area^34^. Rational use of environmental capacity not only can effectively protect the water quality, but also can make the utmost of the dilution and self-purification capacity of the sea area. Therefore, the environmental capacity utilization was selected as the indictor to judge the hydrodynamic environment of the sea area.

(2) Ecological impact indicator

Marine eco-environmental sensitive area refers to the high value sea areas that are difficult to restore after the ecological service function are damaged. They mainly include special protection areas such as the natural reserves, natural concentrated distribution areas for rare and endangered marine life and key fisheries areas, which are relatively sensitive to pollutants discharged. The location of outfalls must be away from the marine ecological sensitive areas. The ecological impact index was selected to evaluate the impact on marine eco-environmental sensitive areas.

#### Calculation and criteria of evaluation indicators

The evaluation indicators were compluted by the hydrodynamics-water quality model.

1. Environmental capacity utilization

Environmental capacity defines as the maximum pollutant load that can be received under the requirements of the water quality standards near the sewage outfall. With the water quality control objective as constraints, the environmental capacity of each outfall was calculated based on the water quality model and response coefficient linear programming method.

The 3D convection–diffusion transport equation of pollutants (Eq. 1) is a linear equation, which makes the pollutant concentration field in the target sea area meet the superposition principle.

1 $$\frac{\partial C}{\partial t}+\frac{\partial }{\partial x}(uc)+\frac{\partial }{\partial y}(vc)+\frac{\partial }{\partial z}(wc)=\frac{\partial }{\partial x}({D}_{x}\frac{\partial C}{\partial x})+\frac{\partial }{\partial y}({D}_{y}\frac{\partial C}{\partial y})+\frac{\partial }{\partial z}({D}_{z}\frac{\partial C}{\partial z})+Q$$

where C is the concentration of a dissolved pollutant, u, v, and w are velocity components, Dx, Dy, and Dz are the diffusion coefficients in the x-, y-, and z-directions, respectively, Q is a source or sink term; and t represents time.

Therefore, in the seawater of the target sea area, it is assumed that the background concentration field formed by the non-point source pollutants and the pollutants input from outside the bay is $C_{0} (x,y,z)$, the pollutant concentration distribution field formed due to the combined action of multiple pollution sources $C(x,y,z)$ shall be equal to the linear superposition of the concentration distribution field formed due to the single action of each pollution source ${C}_{i}(x,y,z)$:

2 $$C(x,y,z)={C}_{0}(x,y,z)+{\sum }_{i=1}^{n}{C}_{i}(x,y,z)$$

The concentration field formed due to the single action of the i pollution source meets:

3 $${C}_{i}(x,y,z)={\alpha }_{i}(x,y,z)\cdot {Q}_{i}$$

where ${Q}_{i}$ is the intensity of i pollution source; ${\alpha }_{i}(x,y,z)$ is a response coefficient field, associated with hydrodynamic condition, topography, etc. The response coefficient is equivalent to the equilibrium concentration field formed by unit source intensity^5^.

The calculation of the maximum allowable quantity of pollutant discharged from outfall can be expressed as the following linear programming problems^35^:

4 $${Q}_{i}=({C}_{i}^{s}-{C}_{i}^{0})/{\alpha }_{i}$$

where ${Q}_{i}$ is the maximum allowable quantity of i pollution source, that is the environmental capacity of i pollution source; ${C}_{i}^{0}$ is the background concentration at the control point, ${C}_{i}^{s}$ is the target concentration of water quality at the control point, and ${\alpha }_{i}$ is the response coefficient.

The environmental capacity utilization (E) was defined as the ratio of the current discharge scale to the environmental capacity of sewage outlet, which can better sort and class the utilization characteristics of marine environmental capacity of each outfall. The formula is calculated as follow:

5 $$E=\frac{{Q}_{i}^{s}}{{Q}_{i}}\times 100\%$$

where ${Q}_{i}$ is the environmental capacity of i pollution source ; ${Q}_{i}^{s}$ is discharge quantity of i pollution source .

The environmental capacity represents the maximum allowable quantity of pollutant discharged from outfall. E is greater than 100%, indicating that the excess pollutants were not accommodated in the area surrounding discharge outfall. At this time, the hydrodynamics condition grade is defined as weak and the score (${E}_{s}$) is assigned to 1. At E was less than 100%, it can be divided into three grades in each partition, namely strong, relatively strong and relatively weak. The hydrodynamic conditions indicator and scoring criteria was shown in Table 2.

(2) Ecological impact index

**Table 2:** Evaluation indicator and criteria of marine hydrodynamic conditions.

Hydrodynamic conditions indicator	Standard	Hydrodynamic grade	Score(${E}_{s}$)
Environmental capacity utilization (E)	E ≤ 30%	Strong	4
	30% < E ≤ 60%	Relatively strong	3
	60% < E ≤ 100%	Relatively weak	2
	E > 100%	Weak	1

The ecological impact index (R) was defined as the ratio of the simulation concentration to the target concentration of water quality at the marine eco-environment sensitive area. The formula is calculated as follow:

6 $$R=\frac{{C}^{p}}{{C}^{s}}\times 100\%$$

where ${C}^{p}$ is the simulation concentration on the marine eco-environment sensitive area based on the 3D water quality model, ${C}^{s}$ is the target concentration of water quality at the marine eco-environment sensitive area.

The discharge pollutants should not damage the Marine eco-environmental sensitive area. R is greater than 100%, suggesting that impact is very large and the ecological impact score (${R}_{s}$) is assigned to 1. In order to sort the impact of each sewage, the ecological impact index was divided into four grades ranked from strong to weak according to the percentage. The indicator and criteria of ecological impact on marine eco-environmental sensitive areas are shown in Table 3.

**Table 3:** Evaluation indicator and criteria of ecological impact for pollutant discharge.

Ecological impact indicator	Standard	Impact grade	Score (${R}_{s}$)
Ecological impact index (R)	R ≤ 30%	Small	4
	30% < R ≤ 60%	Relatively small	3
	60% < R ≤ 100%	Relatively large	2
	R > 100%	Large	1

#### Comprehensive evaluation

The hydrodynamic condition is as important as the ecological impact from the perspective of marine environmental protection and pollution control. Therefore, two indicators have the same weight when evaluating the layout optimization of sewage outfalls. The comprehensive score (S) was defined as the sum with the score of environmental capacity utilization and ecological impact index in order to evaluate the layout and optimize discharge scale of sewage outfalls. The formula is calculated as follow:

7 $$S = E_{s} + R_{s}$$

The optimization objectives are to ensure that there is enough environmental capacity near the outfall to contain pollutants and the discharged sewage should not affect the water quality of the surrounding ecologically sensitive areas. Both of these conditions are indispensable, the comprehensive evaluation scoring criteria for the layout optimization of sewage outfalls was determined. When S ≤ 3, the discharge outlet is defined as irrational. In contrary, when S > 3, the discharge outlet is defined as rational. The comprehensive evaluation criteria is shown in Table 4.

**Table 4:** Comprehensive evaluation criteria for optimization of sewage outfalls.

Comprehensive evaluation	Standard	Evaluation grade
Comprehensive score (S)	S ≤ 3	Irrational
	3 < S ≤ 7	Relatively rational
	S > 7	Rational

### Hydrodynamic-water quality model

In this paper, a 3D hydrodynmic-water quality model for XMB was established by adopting the MIKE3 hydrodynamic module and water quality module. MIKE3 is professional engineering software used to simulate hydrodynamics, water quality and sediment. With advanced pre-processing and post-processing functions and friendly user interface, MIKE3 has been widely applied in studies on estuary, coast and marine^36^. The unstructured triangular mesh was used in XMB model and the mesh was encrypted near the outfall. The number of horizontal meshes was 7,002 and the total number of mesh points was 12,412. The maximum resolution of the mesh was 1.5 km, and the minimum resolution near the outfall was 30 m. The XMB model was divided into five layers by a vertical sigma coordinate system, while the bottom friction coefficient was set 0.01. According the CFL condition and the topography, the time step and the dry–wet critical depth was choose 10 s and 0.1 m, respectively. The XMB topography was shown in Fig. 1.

The model has two open boundaries, the southern boundary and the eastern boundary,which were controlled with the predicted tidal elevation calculated by 11 tidal components (M_2_、S_2_、N_2_、K_2_、K_1_、O_1_、Q_1_、P_1_、M_4_、MS_4_ and M_6_). The data of Jiulong River and other rivers were obtained from the average monthly flow rate in 2018 as the runoff boundary conditions.

In the water quality model, the pollution sources discharged into the sea were generally divided into 17 direct point sources and 11 river pollution sources. The sources locations were shown in Fig. 1. The pollutant loads data were obtained from the 2018 monitoring land-based pollutant sources into the sea measured by the Ocean and Fisheries Bureau of Xiamen (http://www.hyj.xm.gov.cn/Ocean/Index.aspx). Biochemical reaction process of pollutants was not considered in water quality model.

## Results

### Model verification

**Figure 3:**
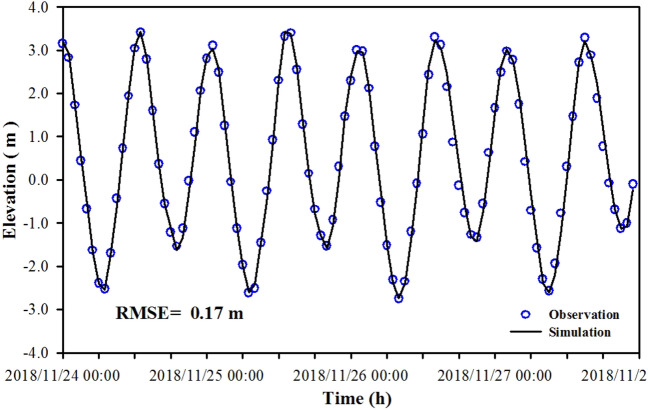
Comparison between the observation and simulation of tidal elevation versus time. The figure was drawn with SigmaPlot Version 10.0.

**Figure 4:**
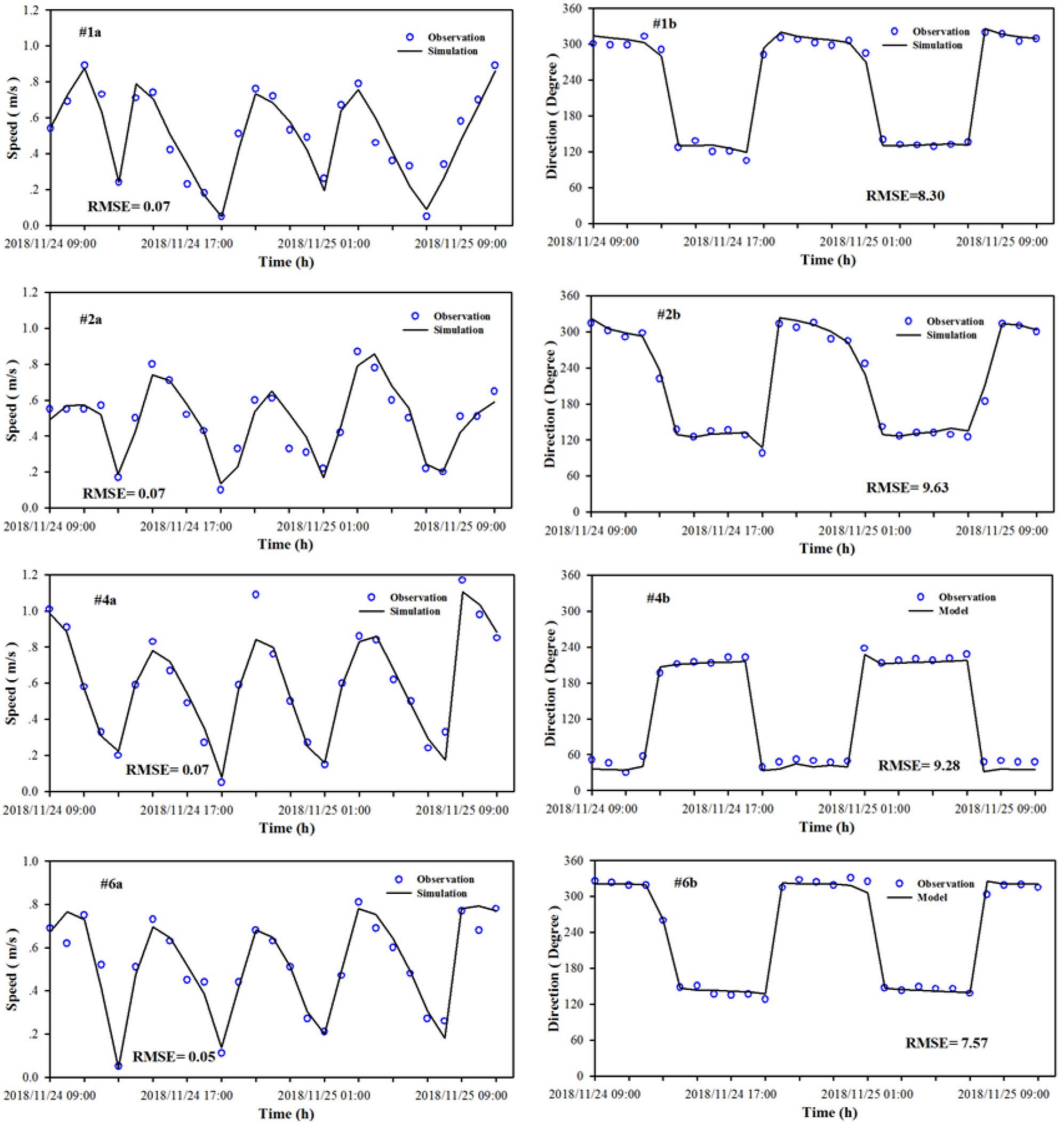
Comparison speed and direction of tidal currents with time between observation and simulation. These figures were prepared with SigmaPlot Version10.0.

The model verification data are obtained from the hydrodynamic monitoring conducted by the Third Institute of Oceanography during the spring tide from November 24 to 25 in 2018, with one tidal elevation station and six tidal current stations (see Fig. 1 for location). Figure 3 shows a verification curve for the tide gauge station of Xiamen Wutong Port. The root mean square error (RMSE) was used for model performance. The comparison between the calculated and the measured tidal level shows that the calculated level agreed with the measured level well. The RMSE of the tidal elevation was 0.17 m, which can better reflect the changing process of the tidal level in XMB. Figure 4 shows the verification results of velocity and direction during the spring tide. The RMSE of the current and direction ranged 0.05–0.07 m and 7.57°–9.52°, respectively. As we seen from the figure that the simulation results of most observation station agreed with the measured values, and the flow regime of each station shows obvious rectilinear current, indicating that the model can better simulate the change of tidal current movement in this area and provide hydrodynamic driving conditions for the water quality model.

The 2018 survey data from the monitoring report on trend of marine environment quality in coastal area Xiamen measured by the Ocean and Fisheries Bureau of Xiamen was used to validated the water quality model. The duration time running the model is about one year. Chemical oxygen demand (COD), dissolved inorganic nitrogen (DIN) and active phosphate (AP) were simulated as independent components in the water quality model. The comparsion between the model results and the observation values was shown in Table 5 (the stations see Fig. 1). The relative error (RE) of COD, DIN and AP ranged from 2.83–10.91%, 3.85–16.18%, 3.70–17.86%. Their average relative error is 6.72%, 10.44% and 9.68%, respectively. The model error is mainly caused by the inaccuracy of pollution load and the model coefficient. Although there are some errors in the model, the observed values are overall in good agreement with the simulated values.

**Table 5:** Comparsion between observation (Obs.) and simulation (Si.) concentration of COD, DIN and AP.

Stations	COD (mg/L)			DIN (mg/L)			AP (mg/L)		
	Obs	Si	RE (%)	Obs	Si	RE (%)	Obs	Si	RE (%)
XM03	1.05	1.13	7.62	0.85	0.89	4.71	0.048	0.042	12.50
XM06	1.1	1.22	10.91	0.68	0.79	16.18	0.036	0.04	11.11
XM07	0.89	0.83	6.74	0.52	0.5	3.85	0.027	0.026	3.70
XM09	1.05	1.1	4.76	0.39	0.34	12.82	0.023	0.022	4.35
XM11	1.29	1.2	6.98	0.48	0.42	12.50	0.028	0.023	17.86
XM12	1.46	1.33	8.90	0.68	0.57	16.18	0.048	0.041	14.58
XM14	0.99	1.04	5.05	0.21	0.22	4.76	0.015	0.016	6.67
XM16	1.06	1.03	2.83	0.24	0.21	12.50	0.015	0.014	6.67

### Results of environmental capacity utilization

According to *2020 Xiamen Ecological Enviromental Quality Bulletin*^37^, the compliance rate of the functional areas was 70.0%, suggesting slightly eutrophicated in this area. The major pollutants in seawater were AP and DIN, which exceeded the second degree of Sea Water Quality Standards. Therefore, COD was selected as the calculation index of environmental capacity in this study. The water quality target value was constrained by the standard value given in the Environmental Function Zoning in Offshore Areas in Fujian Province (Revised) (2011–2020). The environmental capacity utilization (E) of each sewage outfalls was calculated with the model and Eqs. (2)–(5), and the results were shown in Table 6. As we can be seen from Table 6 that:

1. Shizhoutou outfall faces the open sea and has the best hydrodynamic conditions with the maximum COD environmental capacity of 16.4 t/d. Located at the mouth of the bay, Aotou, Chakouyang and Houyu outfalls have good hydrodynamic conditions and large environmental capacity. Due to the location in Tong’an Bay interior, Fenglin outfall has the weakest hydrodynamic condition with the minimum environmental capacity of 2.16 t/d.
2. In 2025, the environmental capacity utilization of Yundang, Shizhoutou, Fenglin and Dalipu outfalls were 61.22%, 60.98%, 83.33% and 66.67% respectively, indicating that there is still a certain pollutant carrying space in these areas. The environmental capacity utilization of Chakouyang and Aotou outfalls was 35.71% and 10.53% respectively, indicating that there is still a large pollutant carrying space in these areas.
3. In 2035, the discharge scale of Haicang and Aotou Wastewater Treatment Plants were expanded, and the environmental capacity utilization was 71.43% and 72.37% respectively. There is still certain pollutant carrying space in the sea areas near the two outfalls. With the expansion of Jimei and Gaoqi Wastewater Treatment Plants, the environmental capacity utilization was 138.89% and 166.67% respectively, exceeding the pollutant carrying capacity of the sea areas. Pollutants will gather near the two outfalls, causing some pollution to these sea areas.

**Table 6:** COD environmental capacity utilization (E) of each outfall in different conditions.

Name	Discharge area	COD environmental capacity(t/d)	Allowable discharge quantity (t/d)	E (%)	
				2025	2035
Yundang Plant	Houyu	9.8	49 (Grade1A)	61.22	61.22
Haicang Plant	Chakouyang	11.2	56 (Grade1A)	35.71	71.43
Qianpu Plant	Shizhoutou	16.4	82 (Grade1A)	60.98	60.98
Jimei Plant	Fenglin	2.16	18 (Class IV)	83.33	138.89
Aotou Plant	Aotou	15.2	76 (Grade1A)	10.53	72.37
Gaoqi Plant	Dalipu	6	30 (Grade1A)	66.67	166.67

### Results of ecological impact index

The ecological sensitive areas of marine environment in Xiamen include the Chinese White Dolphin Nature Reserve (CWDNR) in West Sea and Tong’an Bay estuary, the Egret Nature Reserve (ENR) in Jiyu and Dayu, the Lancelet Nature Reserve (LNR) in Huangcuo area (see Fig. 2 for location ). The water quality target in marine ecological sensitive area implement the first degree of Sea Water Quality Standards according the Environmental Function Zoning in Offshore Areas in Fujian Province (Revised) (2011–2020). The maximum concentration and the ecological impact index were calculated by the water quality model for XMB. The results are shown in Table 7.

**Table 7:** Ecological impact index (R) of each outfall on marine eco-environment sensitive area.

Name	Discharge area	Marine Eco-Environment Sensitive Area	C^s^ (mg/L)	C^p^ (mg/L)		R (%)	
				2025	2035	2025	2035
Yundang Plant	Houyu	CWDNR (West Sea)	2.0	2.50	2.50	125	125
Haicang Plant	Chakouyang	ENR (Jiyu)	2.0	1.37	1.44	68.5	72
Qianpu Plant	Shizhoutou	LNR (Huangcuo sea area)	2.0	1.91	1.91	95.5	95.5
Jimei Plant	Fenglin	CWDNR (Tong’an Bay estuary)	2.0	1.44	1.46	72	73
Aotou Plant	Aotou	CWDNR (Tong’an Bay estuary)	2.0	1.02	1.49	51	74.5
Gaoqi Plant	Dalipu	CWDNR (Tong’an Bay estuary)	2.0	1.73	2.34	86.5	112

The sewage discharged from Houyu outfall would affect the CWDNR in West Sea area. In 2025 and 2035, the ecological impact index will be 125%, which is very significant. This is mainly caused by that the outfall is located in the CWDNR (West Sea).

The sewage discharged from Chakouyang outfall would affect the ENR in Jiyu. In 2025 and 2035, the discharge scale of Yundang plant will be expanded from 20 × 10^4^ to 40 × 10^4^ t/d, and the ecological impact index will be from 68.5 to 72%, with a relatively large impact on the environment.

The sewage discharged from Shizhoutou outfall would affect LNR of Huangcuo sea area. In 2025 and 2035, the discharge scale of Qianpu plant will be expanded to 50 × 10^4^ t/d, and the ecological impact index will be 95.5%. As the distance between the outfall and Lancelet Nature Reserve is about 200 m, with a relatively large impact on the ecological environment.

The sewage discharged from Aotou outfall would affect CWDNR in Tong’an Bay estuary. In 2025, the discharge sacle of Aoto plant will be expanded from 8 × 10^4^ to 55 × 10^4^ t/d, and the ecological impact index will increase from 48 to 74.5%, with a relatively impact on the environment.

The sewage discharged from Dalipu outfall would affect CWDNR in Tong’an Bay estuary. In 2025 and 2035, the discharge scale will be expanded from 20 × 10^4^ to 50 × 10^4^ t/d, and the ecological impact index will be increase from 86.5 to 112%. In 2035, it will have a relatively large ecological impact on the marine environment sensitive area.

### Results of comprehensive evaluation

According to the resuls of environmental capacity utilization and ecological impact index, the comprehensive evaluation results of each sewage outfalls in 2025 and 2035 were calculated, which showed in Tables 8 and 9.

**Table 8:** Comprehensive evaluation of sewage outfalls in XMB in 2025.

Name	Discharge area	E (%)	Hydrodynamic grade	E_s_	R(%)	Impact grade	R_s_	S	Evaluation
Yundang	Houyu	61.22	Relatively weak	2	125	Large	1	3	Irrational
Haicang	Chakouyang	35.71	Relatively strong	3	68.5	Relatively large	2	5	Relatively rational
Qianpu	Shizhoutou	60.98	Relatively weak	2	95.5	Relatively large	2	4	Relatively rational
Jimei	Fenglin	83.33	Relatively weak	2	72	Relatively large	2	4	Relatively rational
Aotou	Aotou	10.53	Strong	4	51	Relatively small	3	7	Relatively rational
Gaoqi	Dalipu	66.67	Relatively weak	2	86.5	Relatively large	2	4	Relatively rational

**Table 9:** Comprehensive evaluation of sewage outfalls in XMB in 2035.

Name	Discharge area	E (%)	Hydrodynamic grade	E_s_	R(%)	Impact grade	R_s_	S	Evaluation
Yundang	Houyu	61.22	Relatively weak	2	125	Large	1	3	Irrational
Haicang	Chakouyang	71.43	Relatively weak	2	72	Relatively large	2	4	Relatively rational
Qianpu	Shizhoutou	60.98	Relatively weak	2	95.5	Relatively large	2	4	Relatively rational
Jimei	Fenglin	138.89	Weak	1	73	Relatively large	2	3	Irrational
Aotou	Aotou	72.37	Relatively weak	2	74.5	Relatively large	2	4	Relatively rational
Gaoqi	Dalipu	166.67	Weak	1	112	Large	1	2	Irrational

As can be seen from Table 8, in 2025, Yundang outfall is located in the middle of Xiamen’s West Sea area, E_s_ is assigned to 2 points when E is 61.22%. R_s_ is assigned to 1 point when R is 125%. The comprehensive evaluation score is calculated to 3 points, evaluated as the sewage outfall with irrational layout. In 2025, the comprehensive score is 5 points for Chakouyang outfall, 4 points for the comprehensive score of Shizhoutou, Fenglin and Dalipu outfalls, and 7 points for Aotou outfall. They are all evaluated as the outfalls with relatively rational layout.

As can be seen from Table 9, in 2035, E_s_ of Shizhoutou outfall is assigned to 2 points when E reaches 60.98%, and R_s_ is assigned to 2 points when R is 95.5%, then the comprehensive evaluation score is 4 points, evaluated as the outfall with relatively rational layout. The discharge scale from Fenglin outfall will be increased to 25 × 10^4^ t/d, and E will reach 138.89%, which will exceed its environmental capacity. The comprehensive evaluation score of the Fenglin outfall is 3 points, evaluated as the outfall with irrational outlay. According to the value of E and R, E_s_ and R_s_ of Dalipu outfall were assigned to 1 point, indicating that the discharge scale would far exceed its environmental capacity and have a large impact on the environment of CWDNR at Tong’an Bay estuary. The comprehensive evaluation score of the Dalipu outfall is 2 points, evaluated as the irrational outfall. In 2035, the comprehensive score of Chakouyang and Aotou outfalls will be 4 points, evaluated as the outfalls with relatively rational layout.

## Discussion and optimization scheme

### Optimization scheme

According to the evaluation results of the layout of sewage outfalls in Xiamen, the following optimization conclusions are proposed:

1. Houyu outfall was evaluated as the outfall with irrational layout comprehensively because it is located in the CWDNR of West Sea area with a relatively large impact on the marine sensitive area. Houyu outfall built in 1997 before the establishment of the marine nature reserve. Considering the hydrodynamic conditions and the ecological sensitivity characteristics of the area, Yundang Wastewater Treatment Plant will remain its treatment capacity unchanged, 30 × 10^4^ t/d. It is suggested to increase the reclaimed water reuse rate or improve the standard in order to reduce the total quantity of pollutant discharged into the West Sea.
2. The Fenglin outfall of Jimei Wastewater Treatment Plant is shallow in depth and has poor hydrodynamic conditions, which can accommodate the maximum sewage discharge scale of 18 × 10^4^ t/d. When the discharge scale will increase to 25 × 10^4^ t/d in 2035, it will exceed its environmental capacity and affect the environmental quality of surrounding sea areas. It is comprehensively evaluated as the irrational outfall due to the large discharge scale, suggesting to control the discharge scale at 18 × 10^4^ t/d in 2035 or improve the reclaimed water reuse rate.
3. The maximum allowable discharge scale of Dalipu outfall of Gaoqi Wastewater Treatment Plant is 30 × 10^4^ t/d. The discharge scale in 2035 will increase to 50 × 10^4^ t/d, which will exceed its environmental capacity and affect the CWDNR of Tong’an Bay estuary. It is proposed that the discharge scale should be controlled at 30 × 10^4^ t/d in 2035, or the discharge standard should be improved, or the reclaimed water reuse rate should be improve by adopting the reconstruction scheme.

### The indicator system

The location of sewage plant outfalls in coastal area is related to the environmental and economic factors according to the actual situation. It is very challenge to construct a comprehensive indicator system that can reflect the current conditions of the optimization selection model. Considering the principle of covering major factors, the construction of the index system focuses on the environmental condition and impact convenience in data availability and in operation. Due to the optimization of existing sewage outfall, the economic conditions were ignored. Through comparing the comprehensive index scores of different sewage outlets, the reasonableness and discharge scale of the sewage outlet were evaluated and optimized, which can provide the guidelines for management decision makers.

It is most important to determine the evaluation criteria and quantify the weight for the indicators in the optimization process after construction of evaluation indicator system. The hydrodynamic condition and the impact on the marine eco-environment sensitive are the same weight, and regarding the same importance of the optimization model. The environmental capacity and the ecological impact are considered the most important standards for the pollution management of sewage outlets into the sea, which is consistent with previous studies^8,23^.

At present, the pollution of China's coastal waters is serious, and the improvement and protection of the sewage outlet into the sea has become a key point of the environmental protection work. The research hot is how to select the appropriate scheme and optimize the existing sewage outlet from a regional perspective. The optimization of sewage outlet involves environmental management issues of environmental, social, economic and other factors. How to make a decision among these often-conflicting attributes is a difficult process. In this study, the evaluation index system is constructed based on the most important Hydrodynamic index and environmental impact factors of the existing sewage outlet. And the optimization of the sewage outlet is carried out by combining with the mathematical model. Although there are some limitations in index selection and index classification in this study, it provides scientific support for the improvement and optimization of the existing sewage outlet.

### The simulation model

A 3D model of hydrodynamics and water quality in XMB was built to calculate the evaluation index values and optimize the sewage outfalls. The time-series observed data of surface elevation and current were compared and analyzed. The water quality state variables such as COD, DIN and AP were also validated in the water quality model.

The environmental capacity utilization and ecological impact index were calculated based on the validated model. The results show that the model can well simulate the transport and diffusion of pollutants in XMB and provide data support for the evaluation system of sewage outlet.

The water quality model in this study simulates COD, DIN and AP as separate variables without considering the biochemical reaction process to evaluate the impact of hydrodynamic conditions and pollutant diffusion on the ecological environment. In subsequent studies, a three-dimensional eutrophication model will be established to simulate the change process among various elements and improve the simulation accuracy. Because the current situation of nitrogen and phosphorus pollutants in XMB exceeds the standard seriously, the environmental capacity of COD was only considered in this study. Indeed, the multiple pollutants should be selected as far as possible for evaluation when this model applied in other marine areas.

## Conclusions

In this paper, from the perspective of marine environmental protection and pollution control in offshore areas, an evaluation method for the layout optimization of sewage outfalls was established. Based on the marine numerical simulation and comprehensive evaluation technology, the rationality of the layout of sewage outfalls was comprehensively evaluated by considering the hydrodynamic conditions and the ecological impact on the marine eco-environment sensitive area.

The layout rationality was evaluated and the discharge scale was optimized with a case study of existing sewage outfalls in Xiamen based on the established method. The results show that, the comprehensive evaluation score of Yundang outfall in 2025 is 3 points, evaluated as the outfall with irrational layout. The comprehensive evaluation scores of Fenglin and Dalipu outfalls are 3 and 2 points respectively in 2035, evaluated as the outfalls with irrational discharge scale. It is suggested to control the discharge scale or increase the reclaimed water reuse rate.

The method for evaluation of layout rationality of sewage outfalls established in this paper takes improvement and protection of the marine environment as its primary objective. Based on the numerical simulation and comprehensive evaluation method, an outfall layout optimization scheme was proposed by combining the local improvement of sewage outfalls layout and the regional sewage discharge scale optimization, providing scientific supports for marine environment management and layout optimization of outfalls. This method focuses on the factors affecting the marine environment. However, it ignores the impact of other factors such as the biological effects (for example, *Escherichia coli*) and project risk of engineering construction, which has certain limitations. In order to meet the requirements of marine governance and management of outfalls in the future, it is necessary to improve the index system and evaluation method established in this paper, so as to make them more operable in practice.
